# Bluetooth Low Energy Beacon Sensors to Document Handheld Magnifier Use at Home by People with Low Vision

**DOI:** 10.3390/s21217065

**Published:** 2021-10-25

**Authors:** Ava K. Bittner, Max Estabrook, Niki Dennis

**Affiliations:** 1 Stein Eye Institute, Department of Ophthalmology, University of California Los Angeles (UCLA), Los Angeles, CA 90095, USA; mestabrook@mednet.ucla.edu; 2 Computing Technologies Research Lab, Health Information Technology, University of California Los Angeles (UCLA), Los Angeles, CA 90095, USA; nfdennis@ucsc.edu

**Keywords:** low vision, visual impairment, vision rehabilitation, magnifier, Bluetooth low energy beacon sensors

## Abstract

We explored the feasibility of using Bluetooth low energy (BLE) beacon sensors to determine when individuals with low vision (LV) use handheld magnifiers at home. Knowing the frequency and duration of magnifier use would be helpful to document increased magnifier use after successful rehabilitation training, or conversely, to know when someone has abandoned a magnifier and requires assistance. Estimote Sticker BLE beacon sensors were attached to the handles of optical handheld magnifiers and dispensed to eight LV subjects to use at home. Temperature and motion data from the BLE beacon sensors were collected every second by a custom mobile application on a nearby smartphone and transmitted to a secure database server. Subjects noted the date and start/end times of their magnifier use in a diary log. Each of the 99 diary-logged self-reports of magnifier use across subjects was associated with BLE beacon sensor recordings of motion (mean 407 instances; SD 365) and increased temperature (mean 0.20 °C per minute; SD 0.16 °C) (mean total magnitude 5.4 °C; SD 2.6 °C). Diary-logged duration of magnifier use (mean 42 min; SD 24) was significantly correlated with instances of motion (*p* < 0.001) and rate of temperature increase (*p* < 0.001) recorded by the BLE beacon sensors. The BLE beacon sensors reliably detected meaningfully increased temperature, coupled with numerous instances of motion, when magnifiers were used for typical reading tasks at home by people with LV.

## 1. Introduction

Bluetooth low energy (BLE) beacon sensors that transmit information about environmental changes can potentially be utilized by healthcare providers to learn about when patients have used a health-related device or aid. In rehabilitation fields, it would be valuable to know whether patients are actively using an assistive device or whether they have abandoned it (i.e., given up) [1,2], in which case additional rehabilitation training and/or reassessment of the patient’s needs would be warranted. Additionally, both researchers and clinicians in rehabilitation fields may be interested to know if patients are using an assistive device more frequently or for longer durations after rehabilitation training to ascertain whether it has been effective for the patient and helpful to improve their outcomes, since providers are not able to accurately predict which patients will have successful outcomes following vision rehabilitation [3]. Even in non-rehabilitation fields, healthcare providers may be interested to know about whether patients are compliant with prescribed interventions, such as eyeglasses [4], eye drops [5], exercise resistance bands [6], toothbrushes [7], test kits [8] or medications [9]. Currently, there is not a validated, efficient system to notify clinical providers or researchers about whether an individual has been using the device that was dispensed or prescribed to them. A potential solution is the use of BLE beacon sensors, which are capable of being attached to healthcare devices or aids.

A thermosensor device that does not involve BLE beacon technology has been previously studied to monitor compliance with spectacle eyeglasses prescriptions [4]. In the field of rehabilitation, it has been reported that BLE patch sensors can be used to measure heart rate, steps and falls in the elderly [10], and BLE beacon sensors that provide localization data have been proposed to track elderly individuals inside of facility buildings [11,12]. For individuals with visual impairment, systems involving BLE beacon sensors are being developed and evaluated to assist with indoor navigation inside buildings [13] or adherence to the use of prescribed eye drops to treat glaucoma [5]. We previously published on our idea to use BLE beacon sensors that measure changes in motion, temperature and/or humidity to track the use of handheld magnification devices (i.e., magnifiers) used by individuals with vision loss, but had only completed preliminary, brief evaluations (up to 150 s) in a clinical office setting [14]. Here, we report on new data collected by BLE beacon sensors that were attached to magnifiers used by visually impaired people in their homes during real-world tasks. We hypothesized that meaningful changes would occur for both motion and temperature at times when magnifiers were used, such that those data, in combination, would be useful indicators of magnifier use by our visually impaired participants, as evidenced by correspondence to self-reported use of the magnifier in diary logs maintained by the participants.

## 2. Materials and Methods

We provided Estimote Sticker BLE beacon sensors to participants in a clinical trial (‘Beacon Sensors and Telerehabiliation to Assess and Improve use of Devices for visual functioning (BeST-AID)’ (NCT04066075) (https://clinicaltrials.gov/ct2/show/NCT04066075 (accessed on 23 August 2021)). Here, we present data collected from eight older adult participants aged 68–93 years who had recently received a new handheld optical magnifier device from their vision rehabilitation provider. Information about each of the participants is provided Table 1. Participants were recruited from three vision rehabilitation practices in the United States: a private optometric practice, Mid-Michigan Eye Care, by Dr. John Kaminski, and two academic centers, the University of California, Los Angeles (UCLA) and University of Nebraska Medical Center, by Drs. Ava Bittner and John Shepherd, respectively. Informed consent was obtained from all participants via phone by a UCLA study coordinator. The multicenter studies received approvals from the Institutional Review Board (IRB) at UCLA.

Each participant received one Estimote Sticker BLE beacon sensor that was attached to the handheld aspect of their light-emitting diode (LED) illuminated magnifier. The BLE beacon sensor was placed either on the top, bottom or side of the magnifier, whichever was in close proximity to their hand while using the magnifier and most comfortable for the participant. When the magnifier was not being used by the participant, the BLE beacon sensor collected data about the ambient temperature in the room, whereas the direct or indirect contact of the participant’s hand and/or fingers with the BLE beacon sensor on the handheld portion of the magnifier was expected to create an increased temperature measurement due to the transfer of body heat from the hand during magnifier use. We anticipated that the BLE beacon sensor would not detect motion when the magnifier was not used, whereas intermittent motion would be detected while participants actively used the magnifier for reading, since it involves making fine movements with the magnifier to scan the reading material.

Along with the BLE beacon sensor, we also provided a loaner smartphone (Samsung Galaxy S6) with a preinstalled mobile application to collect the signals from the transmitting BLE beacon sensors. We developed a dedicated mobile application for this study in order to assign a study ID code to each participant’s BLE beacon sensor data and send the collected data to our secure database server for later analysis. Participants were instructed to keep the loaner smartphone with the study mobile application plugged in and continuously turned on within ten feet of their magnifier use at home. Additionally, they were asked to keep a hand-written diary log for a two-week period to record the days on which they used their magnifier at home, along with the start and end times.

Descriptive data analyses were used to explore the mean, standard deviation (SD) and range of data collected by the BLE beacon sensor and participants’ diary logs. Generalized estimating equations and population-averaged models with logistic regressions and robust variance estimators, with clustering by subject for the repeated measures, were used to explore the relationships between the duration of magnifier use reported in the diary logs and the BLE beacon sensor data for the number of instances of motion recorded as a binary variable, rate of temperature increase and the total magnitude of temperature increase in degrees Celsius (°C). For the comparison of the duration of magnifier use in the diary logs in relation to the rate of temperature increase measured by the beacon sensor, it was necessary to use a transformation of the variable for the rate of temperature increase in order to achieve a more normal distribution for the analyses, as indicated by Box–Cox analysis and tests of the regression residuals. Data were analyzed using Stata/IC version 15.1 (Stata Corp., College Station, TX, USA).

## 3. Results

Each Beacon was configured to dynamically adjust broadcasting intervals from 2600 ms when static to 500 ms upon detecting motion. The BLE beacon’s broadcasting power was set to −12 dBm, which allows for a range of up to 7 m, using a broadcasting frequency range from 2400 MHz to 2483.5 MHz. The recorded frequency of data collection across all participants was 2.05 s, on average (SD 1.87 s; range 0.5–12 s); however, one subject (#3) had data that were collected less frequently than others (for unknown reasons), and the mean frequency was every 1.14 s (SD 0.64; range 0.5–3 s) when this participant was excluded. This analyzed data only accounted for times that recorded motion of the magnifier was detected, so we only measured beacon transmission frequency during periods of usage by the participant.

**Figure 1:**
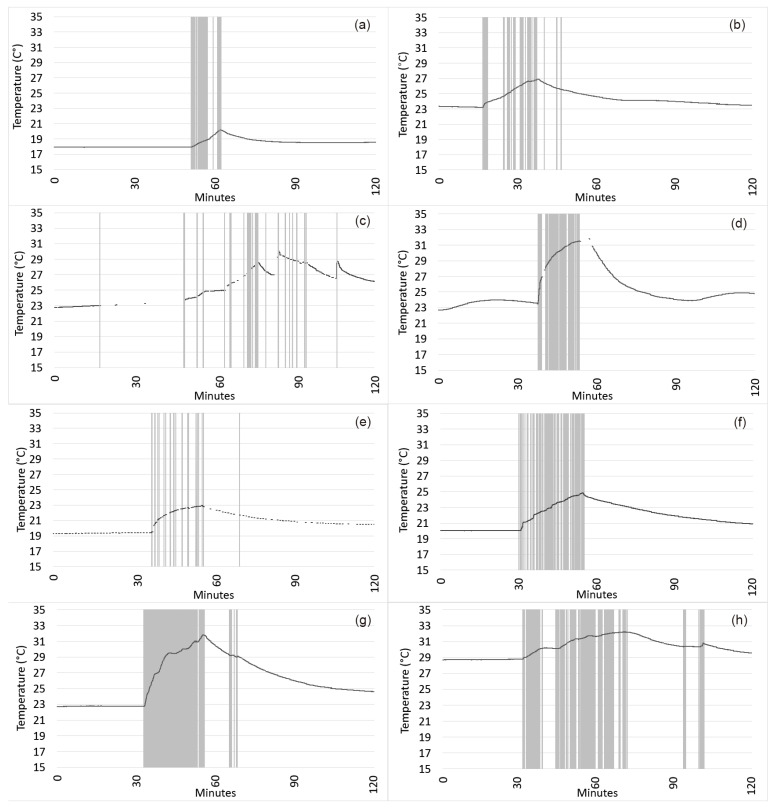
One graph from each participant to display the beacon sensor data for longitudinally collected temperature (black line) and motion, represented by the grey vertical lines, for one diary-logged period for magnifier use, along with additional time shown immediately preceding and subsequent to the magnifier usage. (**a**) Subject 1 reported using the magnifier for 10 min, when the beacon sensor recorded 109 instances of motion and a total temperature increase of 2.25 °C. (**b**) Subject 2 reported using the magnifier for 30 min, when the beacon sensor recorded 209 instances of motion and a total temperature increase of 3.7 °C. (**c**) Subject 3 reported using the magnifier for 30 min, when the beacon sensor recorded 196 instances of motion and a total temperature increase of 4.6 °C. (**d**) Subject 4 reported using the magnifier for 20 min, when the beacon sensor recorded 230 instances of motion and a total temperature increase of 8.3 °C. (**e**) Subject 5 reported using the magnifier for 20 min, when the beacon sensor recorded 372 instances of motion and a total temperature increase of 3.5 °C. (**f**) Subject 6 reported using the magnifier for 24 min, when the beacon sensor recorded 193 instances of motion and a total temperature increase of 4.8 °C. (**g**) Subject 7 reported using the magnifier for 90 min, when the beacon sensor recorded 468 instances of motion and a total temperature increase of 8.8 °C. (**h**) Subject 8 reported using the magnifier for 40 min, when the beacon sensor recorded 447 instances of motion and a total temperature increase of 3.4 °C.

**Figure 2:**
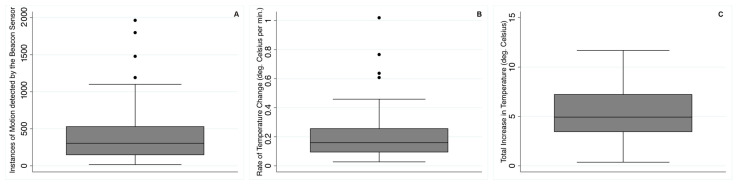
Box plots to show the distribution of the beacon sensor data across all subjects for: (**A**) the number of instances of motion per magnifier use, (**B**) rate of temperature change and (**C**) total temperature rise during each magnifier use. In the box plots, the bottom and top of the box are the 25th and 75th percentile (i.e., the upper and lower quartiles, respectively), and the band near the middle of the box is the 50th percentile (i.e., the median). The ends of the whiskers represent the lowest datum within 1.5 times the interquartile range of the lower quartile and the highest datum still within 1.5 times the interquartile range of the upper quartile. Outlier data are represented by individual dots.

Figure 1 displays representative examples of the results for temperature and motion obtained from the BLE beacon sensor for one time when each participant indicated using their magnifier in the diary log. Note the various patterns of temperature rise according to the duration of magnifier use, as indicated by motion detected by the beacon sensor. Motion appears to be a useful indicator, since all of the 99 self-reports of magnifier use in the diary logs across subjects were associated with a range of 16 to 1965 instances of motion (mean 407 instances of motion; SD 365) detected by the beacon sensor; Figure 2A shows the distribution of the number of instances of motion per self-reported magnifier use. Across all subjects’ data, the beacon sensors measured rapidly increased temperature (0.20 °C per minute on average; range 0.03–1.02 °C, SD 0.16 °C) when several instances of motion were detected, as shown in Figure 2B.

**Figure 3:**
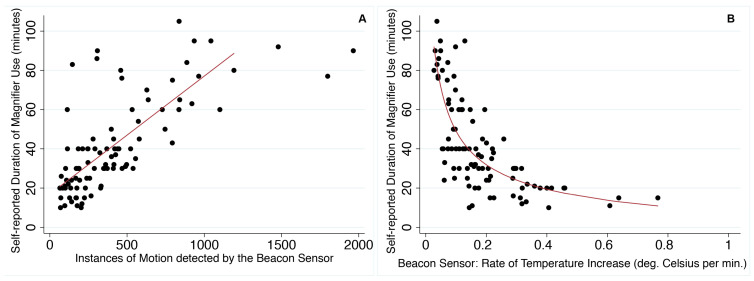
Scatter plots to display the data for the self-reported duration of magnifier use from the diary logs in relation to data recorded by the beacon sensor during the period of magnifier use for: (**A**) the number of instances of detected motion, and (**B**) the mean rate of temperature rise. (**A**) The linear fitted line excludes three outlier data points with greater than 1300 instances of motion. (**B**) The fitted line is a curve for the predicted fractional polynomial.

The self-reported duration of magnifier use in the diary logs (mean 42 min; SD 24, range 10–105 min) was significantly correlated with: (1) the number of instances of motion recorded by the beacon sensor (i.e., self-reported magnifier use increased by 4.4 min, on average, for every 100 instances of detected motion; 95% CI: 2.6, 6.1; *p* < 0.001; Figure 3A) and (2) the mean linear rate of temperature increase measured by the sensor (i.e., self-reported magnifier use decreased by 19.0 min, on average, for every 0.1 °C per minute temperature increase when the rate of temperature increase was <0.3 °C per minute; 95% CI: 9.3, 28.6; *p* < 0.001; Figure 3B; although, there was no statistically significant linear relationship with the amount of beacon sensor-recorded motion when the rate of temperature increase was >0.3 °C per minute, displayed in Figure 3B).

The total magnitude of temperature increase was 5.4 °C, on average, across participants (range 0.4–11.7 °C, SD 2.6 °C) during each period when motion was recorded, as depicted in Figure 2C. The total magnitude of temperature increase did not vary according to the self-reported duration of magnifier use in the diary (*p* = 0.23). The rates of temperature increase during magnifier usage when motion was detected were much greater than the maximum room fluctuations when magnifier use was not reported but due to changes in environmental conditions, e.g., related to indoor heating systems or air conditioning.

## 4. Discussion

In the present study, beacon sensors reliably detected meaningfully increased temperature coupled with several instances of motion during periods when magnifiers were used at home by low-vision (LV) participants during usual, real-world reading tasks. We collected data for a wide range of durations of magnifier use across older adults with LV. Building upon the results of our preliminary study with BLE beacon sensors on magnifiers used by LV patients in clinics [14], the present work provides further support for the continued development and evaluation of similar BLE beacon sensors to develop a system to quantify when magnifiers are used at home. We demonstrated the feasibility of remote, continuous, longitudinal data collection. We propose that both temperature and motion data need to be considered jointly, as the magnifier may detect motion but no temperature increase if bumped inadvertently without use, or conversely, temperature might rise without motion if left in direct sunlight.

As anticipated, our data show a significant positive correlation between self-reported duration of magnifier use and the number of instances of motion detected by the beacon sensor (Figure 3A). We found an inverse relationship between the duration of magnifier use in the diary log and rate of temperature increase recorded by the beacon sensor (Figure 3B). In part, this may be due to the fact that the peak temperature did not necessarily correspond with the end of the magnifier use period (e.g., Figure 1b,c,g,h), as many participants continued to use the magnifier (perhaps intermittently) as the temperature reading declined but the beacon sensor continued to detect motion. Our findings indicate that the fastest temperature rises tended to occur when magnifiers were used for approximately 20 min or less. However, we found a wide range for the rate of temperature increase when magnifiers were used for about 20 min, which might be related to whether the participant’s hand or fingers made direct or indirect contact with the beacon sensor. We did not collect information on direct versus indirect contact with the beacon sensor in this study, but meaningfully increased temperature was measured across all participants, and our previous work indicated that either indirect or direct contact observed in the clinic resulted in increased temperature recorded by the same Estimote sticker BLE beacon sensors [14]. The graphs for temperature, as recorded by the Estimote BLE beacon sensors in the current study, are similar to graphs we previously published for temperature data collected by BlueMaestro BLE beacon sensors attached to handheld magnifiers used by low-vision patients at home [14]. The previously studied BlueMaestro BLE beacon sensors did not collect data on motion and only stored a limited amount of data internally, which does not allow for data collection beyond a few days.

Some advantages of our study design were the inclusion of multiple centers in different regions of the U.S. and data collection during different times of the year to help increase generalizability. The ambient room temperature without magnifier use that was recorded by the BLE beacon sensor varied across participants, and yet, the temperature increases were meaningful across all instances when motion was detected. One inherent limitation in the study design involved the participants’ self-reports of the magnifier use in the daily logs, since they varied in precision for the reported start and end times, as some people were exact, to the minute, for each recorded time, whereas others indicated approximate times. However, the diary logs were helpful to support and confirm that they used the magnifier at that time when the beacon sensor recorded significantly increased temperature and several instances of motion.

To increase compliance with maintaining the diary logs, we tried to limit the burden associated with keeping track of magnifier usage by only asking about the date and start and end times for magnifier use. Therefore, we did not ask the participants to record the type of task for which they used the magnifier. Magnifiers are typically utilized for reading tasks [15], some of which may involve continuous reading, but others can entail intermittent spot reading, possibly combined with some handwriting during the same general time period when the magnifier is used. In our study, it is unknown at which times the magnifiers were used intermittently versus continuously during the time period that was indicated in the diary logs. Future studies could include video recordings of the magnifier usage to distinguish between intermittent versus continuous magnifier use for various types of tasks and then determine relationships with the beacon sensor data for temperature changes and motion. Our data likely contain a variety of magnifier uses, but regardless of whether they were used continuously or sporadically for a task, our data support that both motion and temperature rises occurred in conjunction with magnifier use.

Missing data can occur when the receiving device (i.e., smartphone) is either turned off (or runs out of battery charge) or is out of range of the transmitting device (i.e., BLE beacon sensor). This happens if the two devices are too far away or if there are too many or very dense obstacles in between them. A potential future solution could involve the use of custom development boards that leverage Mesh and LTE cellular capabilities to eliminate the need for BLE robustness while allowing for the customization of data packets and types of sensor data. Future studies should also consider the ergonomics of the placement of the BLE beacon sensor in conjunction with the users’ hands and fingers for comfort and compliance. Additional longitudinal studies in a larger cohort are needed to validate whether BLE beacon sensors can accurately determine when individuals have abandoned their magnifier (i.e., non-usage for an extended period of 1–3 months). In the future, advanced data analysis methods for big data and/or machine learning may be valuable for BLE beacon sensor data in order to distinguish periods of magnifier usage from non-usage. The development of new analytical systems for these data will be important to be able to give alerts to clinical providers if their patients have stopped using their magnifiers over an extended period so that a timely intervention can occur to attempt to remedy any issues, as well as to provide researchers with data on the frequency of magnifier use by study participants to learn more about the longitudinal patterns of magnifier use and their relationship with patient outcomes.

In conclusion, this current work provides further evidence in support of our hypothesis that BLE beacon sensors that detect temperature and motion have the potential to be used as a monitoring system for handheld device usage by individuals at home during usual activities.

## 5. Patents

US provisional patent application: 62/881,257: Systems and Methods for Determining Magnification Device Usage Using Wireless Beacon Sensors (2019).

## Figures and Tables

**Table 1:** The table displays the demographic information and other characteristics of each of the eight study participants.

ID	Site	Ocular	Distance	Age	Gender	Race	Magnifier	Months
#		Diagnosis	Vision(BE)	(Years)				
1	MI	CFH	20/110	68	M	W	5x HHM	Jan.–Feb.
2	MI	AMD	20/63	75	F	W	3.5x SM	December
3	CA	PDR	20/38	70	M	B	3.5x HHM	July
4	MI	AMD	20/65	91	F	W	3.5x HHM	October
5	NE	AMD	20/60	93	F	W	2.5x HHM	March
6	MI	AMD	20/85	73	F	W	3.5x HHM	Dec.–Jan.
7	NE	AMD	20/100	79	F	W	5x HHM	Nov.–Dec.
8	CA	PDR	20/50	70	F	B	4x HHM	Jul.–Aug.

Table Abbreviations: BE = better eye; MI = Michigan: Mid-Michigan Eye Care; CA = California: University of California, Los Angeles; NE = Nebraska: University of Nebraska Medical Center; CFH = congenital foveal hypoplasia; AMD = age-related macular degeneration; PDR = proliferative diabetic retinopathy; F = female; M = male; W = white; B = black or African American; HHM = (illuminated) handheld magnifier; SM = (illuminated) stand magnifier.

## Data Availability

Collected data are housed within an Amazon Web Services (AWS) database. Exports of collected data, along with mobile app source code, are available in a public Github repository.
